# Cross-reactive neutralizing activity in HIV-1 infected injecting drug users

**DOI:** 10.1186/1742-4690-9-S2-P56

**Published:** 2012-09-13

**Authors:** Z Euler, MJ van Gils, H Schuitemaker

**Affiliations:** 1 Academic Medical Center, University of Amsterdam, Amsterdam, the Netherlands

## Background

Vaccine elicited HIV-1 specific cross-reactive neutralizing humoral immunity may provide protection against acquisition of HIV-1. Several studies have focussed on a better understanding of cross-reactive neutralizing activity (CrNA) in natural infection, but only in homosexual and heterosexual HIV-1 transmission cases.

## Methods

To analyze the prevalence and characteristics of CrNA in HIV-1 infected individuals who reported injecting drug use as the only risk factor, we screened serum samples from 50 male and 35 female participants of the Amsterdam Cohort Studies (ACS) on injecting drug users (IDU) for CrNA across a heterologous 6-viral panel. For comparison, similar data from the ACS on men who have sex with men (MSM) were available.

## Results

HIV-1 infected IDU showed significantly lower geometric mean IC50 values and a lower proportion of individuals that was capable of neutralizing the majority of viruses in the panel as compared to MSM. This difference was however no longer observed when women were excluded from the IDU group. Interestingly, three out of 50 in the male IDU population qualified as elite neutralizer as compared to 1 out of 299 among MSM. Multivariate analysis with viral load at setpoint, CD4+ count at setpoint, gender and transmission route as co-variates revealed only CD4+ count at setpoint as independently associated with CrNA in serum.

## Conclusion

CrNA prevalence and potency in HIV-1 infected IDU was lower than in MSM. This could be attributed to the presence of women in the cohort, rather than the route of exposure, which could be explained by the fact that women had a higher CD4+ T cell count at setpoint, which was the only independent predictor for the presence of CrNA. Our data may implicate that the ability of an HIV-1 vaccine to elicit CrNA may be lower in women and testing for gender dependent future vaccine efficacy may be recommended.

